# Environmental parameters to assessing of heat stress in dairy cattle—a review

**DOI:** 10.1007/s00484-018-1629-9

**Published:** 2018-10-27

**Authors:** Piotr Herbut, Sabina Angrecka, Jacek Walczak

**Affiliations:** 1 Department of Rural Building, University of Agriculture in Krakow, al. Mickiewicza 24-28, 30-059 Krakow, Poland; 2 Department of Production Systems and Environment, National Research Institute of Animal Production, 1, Krakowska Street, 32-083 Balice near Krakow, Poland

**Keywords:** Cows, Welfare, Heat stress, THI, Forecasting, Environment

## Abstract

Considering the significant influence of high ambient temperature and heat waves on the well-being and productivity of dairy cows, it is to be expected that, in the course of the next few decades, climate conditions for raising cattle will deteriorate. Research has shown that heat stress causes many negative consequences in terms of physiological and behavioural disturbances and significant losses in milk production. The effort to reduce the risk of the occurrence of heat stress among dairy cows also involves the search for new environmental methods of predicting heat stress. The aim of this paper is to review and systematise the current state of knowledge on the topic of the most widely used environmental methods of determining and predicting heat stress in dairy cows and also to show the directions of studies for the future*.* Based on an analysis of the most popular indexes, the study evaluated their suitability for forecasting heat stress related to maintenance systems and climate conditions for cows. However, the negative results of heat stress often appear with a delay, and a carry-over effect may be experienced (summer heat stress may affect the cows until autumn). The time of the year and breed of cows could have a big impact on when animals become sensitive to increasing heat loads. This likely can be a big contributor to the discrepancies within the different heat stress equations. It is essential to prevent the occurrence of heat stress, predicting it by observing local microclimate conditions and using meteorological forecasts. Thanks to these measures, a breeder may prepare and implement suitable solutions for protecting the animals.

## Introduction

Reports published by international and government research centres indicate an increasing trend towards the systematic warming of the Earth’s climate. Studies conducted by climatologists and meteorologists have shown a particular threat to the whole of Europe (Peltonen-Sainio et al. 2010). These studies suggest that, by 2050, air temperature may rise by as much as 2 °C (Trnka et al. 2011). Bearing in mind the significant influence of heat waves on the well-being and productivity of dairy cows (Cook et al. 2005; De Palo et al. 2006; Herbut et al. 2018), it is to be expected that in the course of the next few decades, climate conditions for raising cattle will deteriorate. This problem will be very significant for cattle breeders throughout the world. In the European Union alone, estimated losses in dairy productivity in 2015 relative to the earlier years totalled between 70 and 550 kg of milk/day for a herd of 100 cows. In 2014, losses estimated using present-day milk prices were US$670 million/year, and this will probably rise to US$2.2 billion/year by the end of the century (Mauger et al. 2015). According to data of the Food and Agriculture Organisation of the United Nations, the USA is one of the largest cow’s milk producers in the world (in 2014, accounting for 14.2% of world production), followed by India, China, Germany and Brazil. Geographic variation is one of the factors affecting the decline in milk production (Mauger et al. 2015). Taking into account the locations in the world of the largest milk producers and the deteriorating heat conditions for cows, the forecasting of heat stress is essential for maintaining global milk production levels.

Reduced milk productivity of cows is considered as the most negative effect of heat stress as its economic results are usually visible after a few days. However, reproductive disorders may be a bigger problem for breeders. Similar like milk yield, the fertility is also depended of heat stress; however, its disorders are more difficult to detect and reveal after a longer period of time. Heat stress makes it difficult to detect oestrus in cows, negatively influences fertility and reduces reproductive capacity by reducing the efficacy of insemination. It also contributes to increase of cases of calving difficulty, postpartum paralysis, increasing the number of stillbirths and the inflammation of the uterine mucus membrane (St-Pierre et al. 2003; Roth 2017). A negative energy balance can also promote ovarian cyst formation, disrupt germinal vesicle development, lead to disturbances in steroid concentration, potentially causing embryonic mortality, and reduce sperm production in stud bulls (De Rensis and Scaramuzzi 2003; Padilla et al. 2006).

Heat stress is defined as the sum of external forces acting on an animal that causes an increase in body temperature and evokes a physiological response (Dikmen and Hansen 2009). It creates the need to meet the requirements of environmental conditions and entails the activation of neuronal and neurohormonal systems, a component of which is the immune system. The degree of the stimulation of these systems determines the intensity of the stress response as well as the consequences they bring to the organism. In that sense, physiological and behavioural disturbances are only mechanisms of adaptation of animals to a threat to the animals’ homeostasis (Adamczyk et al. 2015). Therefore, they are mechanisms to cope with reduced welfare conditions, and they are strictly related to the animals’ wellbeing, so they cannot be considered as a problem themselves, but as stress indicators.

The problem of heat stress is associated with high air temperatures in conjunction with high relative humidity in the animals’ environment (Hill and Wall 2015). In adverse thermal conditions, the animal can dissipate body heat mainly through increased respiration rate, panting, drinking, sweating and reduced feed intake and milk yield. Behavioural coping strategies include increased standing time, shade seeking and decreased activity and movement (De Rensis and Scaramuzzi 2003; Schutz et al. 2009). It is essential to dissipate excess body heat to prevent the animal from entering into a stage of hyperthermia which could have fatal results. Therefore, the maintenance of the correct temperature for the cows is a crucial condition for their high productivity and general health. When the upper critical temperature is exceeded, the adaptive mechanisms of the cows fail to remove the excess heat generated. The occurrence of heat stress may be restricted to one or several days, but it may also extend over a longer period. Other factors influencing the risk of the occurrence of heat stress in cows include the breed of the cow, its age and lactation phase, milk production level, feed and water intake levels, the composition of feed, body condition score and the use of technical solutions to control the animal’s microclimate (Kadzere et al. 2002; West 2003).

Studies conducted in barns and on pasture have shown that favourable environmental conditions, most importantly the correct air temperature and relative humidity range, can be maintained for livestock by applying appropriate solutions (Janni and Allen 2001). There are various cooling options for dairy cows based on the principles of convection, conduction, radiation and evaporation. Other methods include misting and air-mixing devices and water droplets from low-pressure sprinkler systems. Increasingly, often diverse methods of increasing shade coverage are mentioned, including tree coverage, choko vines, roofs, extensions of eaves and the installation of sunlight-reducing mesh, which can create more hospitable microclimates for cows due to the reduction in solar radiation exposure and decline in ambient temperature (Schutz et al. 2009; Angrecka and Herbut 2016; Angrecka et al. 2017).

Indicators of heat stress can be directly measured on the animals (behavioural, physiological, productive and reproductive indicators) and those environmental parameters that can be considered as risk factors. Indicators based only on the environmental parameters can be used to set thresholds, i.e. limits beyond which the risk that animals undergo thermal stress increases. But animals do not necessarily negatively react to the exceeding of these limits. The behavioural response elicited by heat stress can vary based on the breed, age, parity, physiological state, individual characteristics etc.

The effort to reduce the risk of the occurrence of heat stress among dairy cows also involves a search for new methods of predicting heat stress. In order to determine the comfort and heat stress levels of cows, since the 1950s (Thom 1959), scientists have used a variety of indices permitting the increasingly precise determination of the environmental parameters to which the cows are subject.

The aim of this paper is to review and systematise the current state of knowledge on the topic of the most widely used environmental methods of determining and predicting heat stress in dairy cows, and also to show the directions of studies for the future.

### Environmental parameters as risk factor

High productivity among dairy cows coincides with high amounts of metabolic heat production, and this excess heat must be released into the surroundings (Lambertz et al. 2014; Hill and Wall 2015). However, the presence of high temperatures and high relative humidity interferes with this process and the body temperature of the cows rises (Allen et al. 2015), sometimes resulting in inadequate regulation of the cow’s body temperature and the occurrence of heat stress (Rhoads et al. 2009).

Cows are well able to adapt to changeable temperature and humidity conditions throughout the year (Kadzere et al. 2002). This can be confirmed by a relatively wide range of neutral temperatures established for dairy cattle. Fluctuations of temperature within a range of − 0.5 to 20.0 °C and 60–80% relative humidity (West 2003) is generally accepted as a thermoneutral zone that does not significantly induce physiological or behavioural changes among cows. The level of air temperature generally accepted as 25.0–26.0 °C (West 2003) or 24.0–27.0 °C (Brouček et al. 2009) is the upper critical temperature, above which the dairy cow welfare is disturbed. Although air temperature and relative humidity may be most important in determining the exchange of heat between the animal and its surroundings, other relevant microclimate factors, such as air movement and sunlight, also play a significant role in levels of heat stress (Buffington et al. 1981; Shioya et al. 1997; Da Silva et al. 2010). Changes in air velocity influence the convection cooling of cattle which, in combination with solar radiation, has a very significant impact on the regulation of thermal balance of cows (Davis and Mader 2003). The effective air velocity recommended for dairy cattle in the USA during heat stress is from 1.8 to 2.8 m s^−1^ (Bailey et al. 2016). Berman (2005) stated that air velocity was reduced by cows moving in the barn, so that its measurements do not always reflect real values. This is consistent with the research of Herbut et al. (2013), who pointed out the need to perform measurements of air velocity throughout the whole area housing the cows, not just at individual measurement points. It is also worth noting that during the heat period, the use of air velocities above 1.0 m s^−1^ with increased humidity (e.g. through sprinklers) effectively cools cows (Armstrong 1994).

Solar radiation is one of the leading environmental factors that affect livestock. Global radiation includes both direct radiation, which arrives directly from the sun, and the diffuse radiation received from the blue sky and/or reflected by the clouds. The impact of radiation, whether direct, scattered or reflected, may be the main determinant of the environmental conditions in which the cows are kept, primarily as this applies to pastures. For this reason, we assume that cows on pasture are more sensitive to heat stress. This sensitivity also largely depends on breed of cattle, milk yield and pasture management (shelterbelt, shed).

The problem of insolation to a lesser extent can also occur in the case of barns. Studies conducted by Herbut et al. (2015) in free stall barns (loose housing system) showed a significant variation in microclimate conditions caused by the impact of insolation even within the area of the same group of animals. These variations result from the higher air and litter surface temperature experienced during the day in cubicles adjacent to walls that are exposed to solar radiation, as compared to cubicles in shadow (Angrecka and Herbut 2016).

Depending on the cattle system (barn or pasture), the share of the above parameters characterising environmental conditions may have different weight in determining the risk of thermal stress. For example, in the case of pastures, the greatest risk factor is solar radiation, which in cowshed is reduced by the construction of the building. On the other hand, in buildings, the problem is limited effectiveness of natural ventilation and, as a result, the need for mechanical ventilation. For this reason, the assessment of the risk of thermal stress in dairy cattle in the context of various microclimatic parameters should take into account the maintenance system.

### Environmental risk indicators

Over the years, two main methods of assessing environmental risk factors and the animals’ reaction to changing environmental conditions have developed. The first of these are a variety of different temperature-humidity indices expressed in absolute units that define the thermal comfort of the cows with the changing parameters of their environment. The second are algorithms express in °C, which are intended to define the temperature as experienced by the animal. The indices have undergone numerous modifications and feature a variety of different ranges of values defining the extent of heat stress among dairy cows. Many indices have been proposed which are based on measurement of meteorological factors, such as THI (Thom 1959), BGHI (Buffington et al. 1981), ETI (Baeta et al. 1987), HLI (Gaughan et al. 2008), RR (Eigenberg et al. 2005), CCI (Mader et al. 2010), ITSC (Da Silva et al. 2015) and others (Table 1).

**Table 1:** Overview of dairy cow heat stress indices

Index	Name of the index	Authors, publication year
THI	Temperature-humidity index	Thom 1959; NRC 1971
BGHI	Black globe-humidity index	Buffington et al. 1981
ETI	Equivalent temperature index	Baeta et al. 1987
HLI	Heat load index	Gaughan et al. 2003, 2008
THIadj	Adjusted temperature humidity index	Mader et al. 2006
CCI	Comprehensive Climate Index	Mader et al. 2010
ITSC	Index of thermal stress for cows	Da Silva et al. 2015

One index widely used both with cows kept in barns and with cows kept in pastures is THI. This index takes into account the effect of air temperature and humidity, and it is used as a general indicator of heat stress among humans (Thom 1959), in addition to its role in assessing the comfort of dairy cows as well as of other animals, especially livestock. Over the years, the formula for calculating THI has been modified and corrected numerous times (Table 2) by various authors (Steadman 1979; Ravagnolo and Misztal 2000; Mader et al. 2006). An analysis of publications from the last 15 years indicates that the formulas most often used are those developed by Kibler (1964), the National Research Council (1971), and Yousef (1985). Presumably their popularity results from the simplicity of their algorithm and their use of microclimate measurement parameters.

**Table 2:** Formulas for calculating THI values

Authors	Year	Calculation formula
Thom	1959	THI = [0.4 × (Tdb + Twb)] × 1.8 + 32 + 15
Bianca	1962	THI = (0.35 × Tdb + 0.65 × Twb) × 1.8 + 32
Kibler^a^	1964	THI = 1.8Tdb - (1 - RH) (Tdb - 14.3) + 32
National Research Council	1971	THI = (1.8 × Tdb + 32) − (0.55–0.0055 × RH) × (1.8 × Tdb − 26)
Yousef	1985	THI = Tdb + (0.36 × Tdp) + 41.2
Mader et al.	2006	THI = (0.8 × Tdb) + [(RH/100) × (Tdb − 14.4)] + 46.4

*Tdb* dry bulb air temperature, °C, *Twb* wet bulb air temperature, °C, *Tdp* dew point temperature, °C, *RH* relative air humidity, %

^a^In Kibler formula RH is fraction of the unit

The threshold values accepted for the occurrence of heat stress among cows and its influence on productivity have also undergone modification (Hammami et al. 2013). Different authors provide different THI threshold values at which heat stress begins, ranging from 68 to 74 units. According to Du Preez et al. (1990), milk productivity is not affected when THI remains within the range of 35 to 72 units. Hahn et al. (2009) classified levels of heat stress in the following THI ranges: < 74—normal, 75 to 78—alert, 79 to 83—danger, and > 84—emergency. Additionally, they divided the thresholds of occurrence of heat stress depending on the productivity of the cows. For high-yield dairy cows, they assigned a THI value of 72, whereas for low-yield cows, they assigned a value of 74. The studies of other authors, however, indicate THI = 72 as a critical threshold value above which the productive properties of the cows begin to change and a drop in productivity is noted (Ravagnolo and Misztal 2000; Bohmanova et al. 2007; Brouček et al. 2009; Thatcher et al. 2010; Akyuz et al. 2010). Smith et al. (2013) state that when THI is greater than 72, Holstein-Friesian cows experience a reduction in milk production from 35.6 to 34.2 kg/day (− 3.9%). This drop in milk productivity has also been pointed out in studies by Bernabucci et al. (2002), who noted that summer-time milk productivity was lower by 10% than spring-time productivity (29.5 and 26.7 kg/day, respectively). However, the extent to which milk production is affected also depends on traits and parities. Bouraoui et al. (2002) claim that in a Mediterranean climate, Holstein-Friesian cows exhibit a reduced milk productivity and DMI at THI values exceeding 68. Newer studies also indicate 68 THI as the lower limit for the occurrence of heat stress (Segnalini et al. 2011; Carter et al. 2011; Carabano et al. 2014).

The increasing number of days with air temperatures over the critical level for cows brings about an increased threat of heat stress. As Linvill and Pardue (1992) state, an important factor influencing the potential drop in productivity during a 4-day heat period is the total number of hours with values of THI > 74 or THI > 80 on the day preceding the drop in milk productivity. In contrast, Carter et al. (2011) claim that high-yield dairy cows experience heat stress when average daily THI > 68, or when minimum daily THI > 65.

Nevertheless, THI calculation models do not take into account the impact of wind speed or ventilated air movement (Kadzere et al. 2002; West et al. 2003). As Davis and Mader (2003) note, higher speeds of air movement result in the convection cooling of cows during heat waves. These factors may cause that the impact of the THI values will be reduced, and thus positively influence the thermal comfort and milk productivity of the cows (Herbut et al. 2015). For this reason, St-Pierre et al. (2003) developed a correction for THI calculation that determines the decrease of the apparent THI due to the use of the cooling system used for cows in unfavourable weather conditions (Table 3).

**Table 3:** Equations to the reduction of heat in barns for dairy cows at different heat stress level with applied cooling system (based on St-Pierre et al. 2003)

Heat stress level	Equation	Applied cooling system
Moderate	ΔTHI = − 11.06 + (0.25∙Ta) + (0.02∙RH)	System of fans or forced ventilation
High	ΔTHI = − 17.6 + (0.36∙Ta) + (0.04 RH)	Combination of fans and sprinklers
Intensity	ΔTHI = − 11.7 − (0.16∙Ta) + (0.18∙RH)	High-pressure evaporative cooling system

*ΔTHI* decrease of the apparent THI due to the use of the cooling system, *Ta* ambient air temperature, °C, *RH* ambient relative air humidity, %

In conjunction with the trend which has been increasing since the 1990s of keeping cattle in barns, these earlier methods have been applied to the assessment of microclimatic conditions found in housing conditions (Baeta et al. 1987).

The model proposed by some authors for calculating Equivalent Temperature Index was given as follows:

$$ \mathrm{ETI}=27.88-0.456\bullet \mathrm{Ta}+0.010754\bullet {\mathrm{Ta}}^2-0.4905\bullet \mathrm{RH}+0.00088\bullet {\mathrm{RH}}^2+1.1507\bullet \mathrm{V}-0.126447\bullet {\mathrm{V}}^2+0.019876\bullet \mathrm{Ta}\bullet \mathrm{RH}-0.046313\bullet \mathrm{Ta}\bullet \mathrm{V}, $$

where

Ta: air temperature, °C
RH: relative air humidity, %
V: air velocity, m/s

However, the formula does not take into account exposure to direct solar radiation, which may have a significant impact on the environmental conditions within the barn and on the occurrence of heat stress. The algorithm presented here and the results of studies indicate the temperature as experienced by a cow kept primarily in a closed barn. According to Da Silva and Maia (2013), this formula is not very effective in a temperate climate. A similar opinion is held by Lacetera et al. (2003) and Hahn et al. (2009), who additionally note that the ETI formula based on tests and results obtained in climate chambers does not reflect the real production conditions prevalent in pastures and in curtain-sided barns.

One indicator which takes into account the movement of air and the impact of solar radiation is LWSI (The Livestock Weather Safety Index), created in 1970 and first used by the US National Weather Service. Its ideas are further developed in THIadj as defined by Mader et al. (2006) in relation to the panting score of cows and produced the following formula:

$$ \mathrm{THIadj}=4.51+\mathrm{THI}-\left(1.922\bullet \mathrm{V}\right)+\left(0.0068\bullet \mathrm{SR}\right), $$

where

THI: temperature humidity index, −
V: air velocity, m/s
SR: intensity of solar radiation, W/m^2^

The authors of this indicator made reference to various conditions to be found in cow husbandry in pastures, including under sheds or shed roofs, near lines of shade-providing trees in open fields and in unprotected open areas exposed to direct sunlight (Mader et al. 2006). It is important to note that in contrast to normal THI, THIadj also takes into account biological differences among cows, including breed and coat colour. THIadj assumes a lower limit for the occurrence of heat stress as 74, while values from 75 to 78 indicate the alert stage, from 79 to 83 danger conditions, and >84 emergency conditions (Mader et al. 2006; Hahn et al. 2009; Arias and Mader 2010).

Curtain-sided barns are highly specific livestock maintenance buildings, usually equipped with natural gravitational ventilation assisted by mechanical ventilation. The compartments of the barn separate the cattle, reducing and stabilising temperature and relative humidity. However, they are built of constructions of posts, walls and dividers that may significantly block the free flow of air necessary in summer for cooling the cows (Shoshani and Hetzroni 2013). As studies conducted in summer by Herbut and Angrecka (2013) confirm, the use of the THIadj index as defined by Mader et al. (2006) may be justified in the case of curtain-sided barns.

In 2005, Eigenberg et al. created a respiration rate estimator based on research that takes account in its calculations the same air parameters as THIadj. For unshaded areas and an air temperature of > 25 °C, the formula is as follows:

$$ \mathrm{RR}=5.1\bullet \mathrm{Ta}+0.58\bullet \mathrm{RH}-1.7\bullet \mathrm{V}+0.039\bullet \mathrm{SR}-105.7, $$

where

Ta: air temperature, °C
RH: relative air humidity, %
V: air velocity, m∙s^−1^
SR: intensity of solar radiation, W∙m^−2^

Along with an increase in air temperature, the respiration rate of cows in unshaded areas was three times higher than that of cows in shaded areas. The conducted studies also determined that a change in THI values of 1 unit caused a change in respiration of 2 breaths/min (Collier et al. 2006) or 4 breaths/min (Eigenberg et al. 2005). With regard to THI, the authors defined threshold values for respiration rate.

Studies on defining environmental conditions for cows kept in pastures have shown discrepancies caused by failure to account for radiant heat load. To rectify this failure, the Black Globe Humidity Index (Buffington et al. 1981) was developed as follows:

$$ \mathrm{BGHI}=\mathrm{Tbg}+0.36\bullet \mathrm{Tdp}+41.5, $$

where

Tbg: black globe temperature, °C (black globe temperature is measured using a black globe temperature sensor which includes a black globe with a thermometer inserted in the centre)
Tdp: dew point temperature, °C

The authors of this model suggest that environmental conditions expressed with values of less than 70 BGHI units do not significantly influence the well-being of dairy cows. However, at values greater than 75, feed intake begins to be reduced. Buffington et al. (1981) showed a relation between an increase in BGHI and a decrease in milk production among cows kept in areas lacking shade. On the other hand, Da Silva et al. (2007) showed the limited applicability of BGHI in determining heat stress in areas with a tropical climate.

The unreliability of using air temperature to predict heat stress was also noted by Gaughan et al. (2003), who defined the Heat Load Index as follows:

for Tbg < 25 °C

$$ \mathrm{HLI}=10.66+0.28\bullet \mathrm{RH}+1.3\bullet \mathrm{Tbg}-\mathrm{V}, $$

for Tbg > 25 °C

$$ \mathrm{HLI}=8.62+0.38\bullet \mathrm{RH}+1.55\bullet \mathrm{Tbg}-0.5\bullet \mathrm{V}+{\mathrm{e}}^{2.4-\mathrm{V}}, $$

where

RH: relative air humidity, %
V: air velocity, m/s
Tbg: black globe temperature, °C

In order to calculate HLI, it is necessary to take into account relative air humidity and air velocity, and instead of air temperature, black globe temperature was introduced. HLI was developed in order to determine heat stress in beef cattle kept in pastures in Australia (Da Silva and Maia 2013), for air temperatures within the range of 8–45 °C. Initially, only one general formula was used, but in 2008, it was made more precise by defining conditions for the application of two formulas for a black globe temperature of 25 °C (Gaughan et al. 2008). The authors of the HLI define thermoneutral conditions as HLI ≤ 70. Above 70 units, heat stress of varying degrees of severity occurs: from 70.1 to 77—warm, 77.1 to 86—hot and above 86—very hot.

Heat Load Index, apart from wind-chill, was the basis for determining CCI—Comprehensive Climate Index (Mader et al. 2010). CCI can be applied within a range of − 30 to + 45 °C, and thus can be used to determine temperature stress among cows both in very hot and very cold conditions.

Calculations of CCI involve adding corrections to ambient temperature, due to relative humidity, wind speed and sun exposure, permitting the determination of temperature as experienced by the cow. Thanks to this, by using only one index, it is possible to determine the positive or negative impacts of environmental parameters depending on the season (Table 4). Although CCI is a comprehensive tool, it is difficult to find scientific publications describing its application. This may be due to the complicated calculation formulas for the compensation corrections proposed to the model.

**Table 4:** Threshold values of heat stress among cows based on CCI (based on Mader et al. 2010)

Environment conditions	Animal susceptibility		
	Hot conditions	Cold conditions	
		High	Low
No stress	< 25	> 5	> 0
Mild	25 to 30	0 to 5	− 10 to 0
Moderate	> 30 to 35	< 0 to − 5	< − 10 to − 20
Severe	> 35 to 40	< − 5 to − 10	< − 20 to − 30
Extreme	> 40 to 45	< − 10 to − 15	< − 30 to − 40
Extreme danger	> 45	< − 15	< − 40

The ability of the animals to deal with heat stress resulting from solar radiation depends on the physical characteristics of their skin and coat (Hillman et al. 2001; Da Silva et al. 2003). This is all the more true when we consider that in certain cases (tropical regions), the temperature of the surroundings (e.g. the ground, elements of buildings, fences) during the day is usually considerably higher than the air temperature, a fact that the commonly used THI indices do not reflect. For this reason, Da Silva et al. (2015) proposed the ITSC—index for tropical regions, as follows:

$$ \mathrm{ITSC}=77.1747+4.8327\bullet \mathrm{Ta}-34.8189\bullet \mathrm{V}+118.6981\bullet \mathrm{Pv}-14.7956\bullet {\mathrm{Pv}}^2-0.1059\bullet \mathrm{ERHL}, $$

where

Ta: air temperature, °C
V: air velocity, m/s
ERHL: effective radiation heat load, W/m^2^
Pv: partial vapour pressure, kPa

ITSC takes into account the majority of factors relating to exposure to solar radiation.

A comparison of the most popular indexes to evaluate their suitability for forecasting heat stress in dairy cattle is shown in Table 5.

**Table 5:** Application and components of equations for the calculation of the indexes

Index	Parameters						Maintenance system		Region of use	
	Ta	RH	V	SR	Tdp	Tbg	Barn	Pasture	Tropical	Moderate
THI*	x	x			x		x	x	x	x
CCI	x	x	x	x			x	x	x	x
THIadj	x	x	x	x			x	x	x	x
ETI	x	x	x				x		x	
HLI		x	x			x		x	x	
BGHI					x	x		x	x	x
ITSC^b^	x		x					x	x	

*Ta* air temperature, °C, *RH* relative air humidity, %, *V* air velocity, m/s, *SR* intensity of solar radiation, W/m^2^, *Tdp* dew point temperature, °C, *Tbg* black globe temperature, °C

^a^Different configuration of parameters: Ta with RH or Tdp

^b^ITSC additionally comprises: ERHL—effective radiation heat load, W/m^−2^ and Pv—partial vapour pressure, kPa

## Conclusions

Early forecasting of heat stress risk makes it possible to limit its negative impact on cow welfare. Therefore, welfare measurements should be based on environmental indices of heat stress, the animal’s response in coping with difficulties, and on signs that coping effects to maintain homoeothermic conditions are failing. Recognition of heat stress may be generally based on observable clinical symptoms among cows that appear together with high air temperature and defined levels of relative humidity. In order to maintain dairy cattle’s healthiness and performance, it is more important to keep the air temperature at a constant level, or to provide adequately long rest periods in lower temperatures with an efficiently functioning ventilation system placed above the cows to cool them, using the increased velocity of the ventilation air or wind speed than the air temperature itself.

The THI value and that of other indexes is usually the main determinant for heat stress management decisions by the breeder. Moreover, the categorical THI values described above can act as a rough indicator for the effects of heat stress on production measures. THI formulas that determine the environmental risk factors for cows are unfortunately still imperfect because they take into account only factors that shape the microclimate of the air. Other indicators of cow response do not include, for example, the role of the floor (ground) in animal cooling. Since cows spend 8–16 h a day in a lying position, at which time 20–30% of their body surface comes into contact with the ground, it will be necessary to develop a THI of the surface on which the cow is lying. In relation to this, it would also seem advisable to extend the scope of research on ground and floors with regard to their heat exchange properties and role in the cooling of cows.

Oftentimes, however, the negative results of heat stress appear only later, and a carry-over effect may be experienced (summer heat stress may affect the cows until autumn). Thus, the time of year and breed of cow can have a big impact on when animals become sensitive to heat stress. This can contribute to discrepancies in the results for the different equations.

It is essential to prevent the occurrence of heat stress, predicting it by observing and measurements local microclimate conditions and using meteorological forecasts. Using historical collected herd data such as cows’ drop in milk yield, and their analysis in different environmental conditions, especially in the summer, could also help to prevent or mitigate the effect of heat stress. Thanks to these measures, a breeder may prepare and implement suitable solutions in order to protect the animals.

## References

[CR1] Adamczyk K, Górecka-Bruzda A, Nowicki J, Gumułka M, Molik E, Schwarz T, Earley B, Klocek C (2015). Perception of environment in farm animals – A review. Ann Anim Sci.

[CR2] Akyuz A, Boyaci S, Cayli A (2010). Determination of critical period for dairy cows using temperature humidity index. J Anim Vet Adv.

[CR3] Allen JD, Hall LW, Collier RJ, Smith JF (2015). Effect of core body temperature, time of day, and climate conditions on behavioral patterns of lactating dairy cows experiencing mild to moderate heat stress. J Dairy Sci.

[CR4] Angrecka S, Herbut P (2016). Impact of barn orientation on insolation and temperature of stalls surface. Ann Anim Sci.

[CR5] Angrecka S, Herbut P, Nawalany G, Sokołowski P (2017). The impact of localization and barn type on insolation of sidewall stalls during summer. J Ecol Eng.

[CR6] Arias RA, Mader TL (2010). Determination of potential risk of heat stress of cattle in four locations of central and southern Chile. Arch Med Vet.

[CR7] Armstrong DV (1994). Heat stress interaction with shade and cooling. J Dairy Sci.

[CR8] Baeta FC, Meador NF, Shanklin MD, Johnson HD (1987) Equivalent temperature index at temperatures above the thermoneutral for lactating dairy cows. ASAE Paper No. 874015. Amer Soc Agric Engr (ASAE), St. Joseph

[CR9] Bailey T, Sheets J, McClary D, Smith S, Bridges A (2016) Heat abatement. Elanco Dairy Business Unit. https://assets.ctfassets.net

[CR10] Berman A (2005). Estimates of heat stress relief needs for Holstein dairy cows. J Anim Sci.

[CR11] Bernabucci U, Lacetera N, Ronchi B, Nardone A (2002). Effects of the hot season on milk protein fractions in Holstein cows. Anim Res.

[CR12] Bohmanova J, Misztal I, Cole JB (2007). Temperature-humidity indices as indicators of milk production losses due to heat stress. J Dairy Sci.

[CR13] Bouraoui R, Lahmar M, Majdoub A, Djemali M, Belyea R (2002). The relationship of temperature-humidity index with milk production of dairy cows in a Mediterranean climate. Anim Res.

[CR14] Brouček J, Novák P, Vokřálová J, Šoch M, Kišac P, Uhrinčať M (2009). Effect of high temperature on milk production of cows from free-stall housing with natural ventilation. Slovak J Anim Sci.

[CR15] Buffington DE, Collazo-Arocho A, Canton GH, Pitt D, Thatcher WW, Collier RJ (1981). Black globe-humidity index (BGHI) as comfort equation for dairy cows. Trans ASAE.

[CR16] Carabano MJ, Bachagha K, Ramon M, Diaz C (2014). Modeling heat stress effect on Holstein cows under hot and dry conditions: selection tools. J Dairy Sci.

[CR17] Carter BH, Friend TH, Sawyer JA, Garey SM, Alexander MB, Carter MJ, Tomaszewski MA (2011). Effect of feed-bunk sprinklers on attendance at unshaded feed bunks in drylot dairies. Prof Anim Sci.

[CR18] Collier RJ, Dahl GE, Van Baale MJ (2006). Major advances associated with environmental effects on dairy cattle. J Dairy Sci.

[CR19] Cook NB, Bennett TB, Nordlund KV (2005). Monitoring indices of cow comfort in free-stall-housed dairy herds. J Dairy Sci.

[CR20] Da Silva RG, Maia ASC (2013). Principles of animal biometeorology.

[CR21] Da Silva RG, La Scala N, Tonhati H (2003). Radiative properties of the skin and haircoat of cattle and other animals. Trans ASAE.

[CR22] Da Silva RG, Morais DAVF, Guilhermino MM (2007). Evaluation of stress indexes for dairy cows in tropical regions. R Bras Zootec.

[CR23] Da Silva RG, Guilhermino MM, Morais DAEF (2010). Thermal radiation absorbed by dairy cows in pasture. Int J Biometeorol.

[CR24] Da Silva RG, Maia AS, de Macedo Costa LL (2015). Index of thermal stress for cows (ITSC) under high solar radiation in tropical environments. Int J Biometeorol.

[CR25] Davis S, Mader TL(2003) Adjustments for Wind Speed and Solar Radiation to the Temperature-Humidity Index. Nebr Beef Cattle Rep 224 https://digitalcommons.unl.edu/animalscinbcr/224

[CR26] De Palo P, Tateo A, Zezza F, Corrente M, Centoducati P (2006). Influence of free-stall flooring on comfort and hygiene of dairy cows during warm climatic conditions. J Dairy Sci.

[CR27] De Rensis F, Scaramuzzi RJ (2003). Heat stress and seasonal effects on reproduction in the dairy cow—a review. Theriogenology.

[CR28] Dikmen S, Hansen PJ (2009). Is the temperature-humidity index the best indicator of heat stressin lactating dairy cows in a subtropical environment?. J Dairy Sci.

[CR29] Du Preez JH, Hatting PJ, Giesecke WH, Eisenberg BE (1990). Heat stress in dairy cattle and other livestock under southern African conditions. III. Monthly temperature-humidity index mean values and their significance in the performance of dairy cattle. Onderstepoort J Vet Res.

[CR30] Eigenberg RA, Brown-Brandl TM, Nienaber JA, Hahn GL (2005). Dynamic response indicators of heat stress in shaded and non-shaded feedlot cattle, part 2: predictive relationships. Biosyst Eng.

[CR31] Gaughan JB, Goopy J, Spark J (2003) Excessive heat load index for feedlot cattle. Final report prepared for Meat and Livestock Australia Ltd. Australia https://data.globalchange.gov

[CR32] Gaughan JB, Mader TL, Holt SM, Lisle A (2008). A new heat load index for feedlot cattle. J Anim Sci.

[CR33] Hahn GL, Gaughan JB, Mader TL, Eigenberg RA, De Shazer JA (2009). Chapter 5: thermal indices and their applications for livestock environments. Livestock energetics and thermal environment management.

[CR34] Hammami H, Bormann J, M’Hamdi N, Montaldo HH, Gengler N (2013). Evaluation of heat stress effects on production traits and somatic cell score of Holsteins in a temperate environment. J Dairy Sci.

[CR35] Herbut P, Angrecka S (2013). Forecasting heat stress in dairy cattle in selected barn zones with the help of THI and THIadj indexes. Ann Anim Sci.

[CR36] Herbut P, Angrecka S, Nawalany G (2013). Influence of wind on air movement in a free stall barn during the summer period. Ann Anim Sci.

[CR37] Herbut P, Bieda W, Angrecka S (2015). Influence of hygrothermal conditions on milk production in a free stall barn during hot weather. Anim Sci Pap Rep.

[CR38] Herbut P, Angrecka S, Godyń D (2018). Effect of the duration of high air temperature on cow's milking performance in moderate climate conditions. Ann Anim Sci.

[CR39] Hill DL, Wall E (2015). Dairy cattle in a temperate climate: the effects of weather on milk yield and composition depend on management. Animal.

[CR40] Hillman PE, Lee CN, Carpenter JR, Baek KS, Parkhurst A (2001) Impact of hair color on thermoregulation of dairy cows to direct sunlight. ASAE Paper 014031, St. Joseph

[CR41] Janni KA, Allen DM (2001) Thermal environmental conditions in curtain sided naturally ventilated dairy freestall barns. ASABE Paper, ASAE, St. Joseph doi:10.13031/2013.7093

[CR42] Kadzere CT, Murphy MR, Silanikove N, Maltz E (2002). Heat stress in lactating dairy cows: a review. Livest Prod Sci.

[CR43] Kibler HH (1964) Environmental physiology and shelter engineering with special reference to domestic animals. LXVII. Thermal effects of various temperature-humidity combinations on Holstein cattle as measured by eight physiological responses. Research bulletin University of Missouri

[CR44] Lacetera N, Bernabucci U, Ronchi B, Nardone A, Lacetera N (2003). Physiological and productive consequences of heat stress. The case of dairy ruminants. Interactions between climate and animal production.

[CR45] Lambertz C, Sanker C, Gauly M (2014). Climatic effects on milk production traits and somatic cell score in lactating Holstein-Friesian cows in different housing systems. J Dairy Sci.

[CR46] Linvill DE, Pardue FE (1992). Heat stress and milk production in the South Carolina Coastal Plains. J Dairy Sci.

[CR47] Mader TL, Davis MS, Brown-Brandl T (2006). Environmental factors influencing heat stress in feedlot cattle. J Dairy Sci.

[CR48] Mader TL, Johnson LJ, Gaughan JB (2010). A comprehensive index for assessing environmental stress in animals. J Anim Sci.

[CR49] Mauger G, Bauman Y, Nennich T, Salathé E (2015). Impacts of climate change on milk production in the United States. Prof Geogr.

[CR50] National Research Council (1971). A guide to environmental research on animals.

[CR51] Padilla L, Matsui T, Kamiya Y, Kamiya M, Tanaka M, Yano H (2006). Heat stress decreases plasma vitamin C concentration in lactating cows. Livest Sci.

[CR52] Peltonen-Sainio P, Jauhiainena L, Trnka M (2010). Coincidence of variation in yield and climate in Europe. Agric Ecosyst Environ.

[CR53] Ravagnolo O, Misztal I (2000). Genetic component of heat stress in dairy cattle, parameter estimation. J Dairy Sci.

[CR54] Rhoads ML, Rhoads RP, Van Baale J, Collier RJ, Sanders SR, Weber WJ, Crooker BA, Schutz KE, Rogers AR, Cox NR, Tucker CB (2009). Dairy cows prefer shade that offers greater protection against solar radiation in summer: shade use, behaviour, and body temperature. Appl Anim Behav Sci.

[CR55] Roth Z (2017). Effect of heat stress on reproduction in dairy cows: insights into the cellular and molecular responses of the oocyte. Annu Rev Anim Biosci.

[CR56] Schutz KE, Rogers AR, Cox NR, Tucker CB (2009). Dairy cows prefer shade that offers greater protection against solar radiation in summer: shade use, behaviour, and body temperature. Appl Anim Behav Sci.

[CR57] Segnalini M, Nardone A, Bernabucci U, Vitali A, Ronchi B, Lacetera N (2011). Dynamics of the temperature-humidity index in the Mediterranean basin. Int J Biometeorol.

[CR58] Shioya S, Terada F, Iwama Y (1997). Physiological responses of lactating dairy cows under hot environments. Eiyoseirikenkyukaiho.

[CR59] Shoshani E, Hetzroni A (2013). Optimal barn characteristics for high-yielding Holstein cows as derived by a new heat-stress model. Animal.

[CR60] Smith DL, Smith T, Rude BJ, Ward SH (2013). Short communication: comparison of the effects of heat stress on milk and component yields and somatic cell score in Holstein and Jersey cows. J Dairy Sci.

[CR61] Steadman RG (1979). The assessment of sultriness. Part I: a temperature-humidity index based on human physiology and clothing science. J Appl Meteorol.

[CR62] St-Pierre NR, Cobanov B, Schnitkey G (2003). Economic losses from heat stress by US livestock industries. J Dairy Sci.

[CR63] Thatcher WW, Flamenbaum I, Block J, Bilby TR (2010) Interrelationships of heat stress and reproduction in lactating dairy cows. The High Plains Dairy Conference, Amarillo https://pdfs.semanticscholar.org

[CR64] Thom EC (1959). The discomfort index. Weatherwise.

[CR65] Trnka M, Olesen JE, Kersebaums KC, Skjelvag AO, Eitzinger J, Seguin B, Peltonen - Sainio P, Rötter R, Iglesias A, Orlandini S, Dubrovský M, Hlavinka P, Balek J, Eckersten H, Cloppet E, Calanca P, Gobin A, Vučetić V, Nejedlik P, Kumar S, Lalic B, Mestre A, Rossi F, Kozyra J, Alexandrov V, Semerádová D, Žalud Z (2011). Agroclimatic conditions in Europe under climate change. Glob Change Biol.

[CR66] West JW (2003). Effects of heat-stress on production in dairy cattle. J Dairy Sci.

[CR67] West JW, Mullinix BG, Bernard JK (2003). Effects of hot, humid weather on milk temperature, dry matter intake and milk yield of lactating dairy cows. J Dairy Sci.

[CR68] Yousef MK (1985). Stress physiology in livestock. Volume I. basic principles.

